# PPARα modulation of macrophage polarization and inflammatory signaling in mimic periodontitis

**DOI:** 10.1590/1678-7757-2025-0338

**Published:** 2025-10-20

**Authors:** HU Arthur, Yvette Y CHEN

**Affiliations:** 1 Massachusetts Institute of Technology Cambridge United States Massachusetts Institute of Technology, Cambridge, United States.; 2 The ADA Forsyth Institute Department of Immunology and Infectious Diseases Somerville United States The ADA Forsyth Institute, Department of Immunology and Infectious Diseases, Somerville, United States.

**Keywords:** Macrophage, Periodontitis, Porphyromonas gingivalis, Flow cytometry, Lypopolysaccharides

## Abstract

**Objective:**

This study investigates the role of peroxisome proliferator-activated receptor alpha (PPARα) in regulating macrophage polarization and inflammatory signaling under stimulation by periodontal pathogens.

**Methodology:**

THP-1-derived macrophages were stimulated with Porphyromonas gingivalis lipopolysaccharide (Pg-LPS) in the presence or absence of PPARα agonists fenofibrate and WY14643, or the antagonist GW6471. Protein expression levels of TNF-α, IL-10, and phosphorylated NF-κB were assessed by Western blot. Immunofluorescence staining was used to evaluate IL-10, NF-κB, and CD36 expression. Flow cytometry quantified changes in macrophage polarization markers, including CD14^+^CD86^+^ (M1) and CD68^+^CD206^+^/CD163^+^ (M2) populations. THP-1 cells transfected with a secreted embryonic alkaline phosphatase (SEAP) reporter plasmid were treated with Pg-LPS (1 μg/mL) ± fenofibrate (50 μM) to assess NF-κB/AP-1 activity. PPARα reporter cells were treated with increasing concentrations of GW590735 or WY14643 and exposed to TNF-α, LPS, or GW6471+LPS to evaluate PPARα transcriptional activity.

**Results:**

PPARα activation by fenofibrate reduced TNF-α expression in Pg-LPS-stimulated macrophages and attenuated NF-κB signaling via both TLR2 and TLR4 pathways. Fenofibrate significantly increased IL-10 and CD36 expression, inhibited Pg-LPS-induced NF-κB nuclear translocation, and promoted a phenotypic shift from pro-inflammatory M1 to anti-inflammatory M2 macrophages. Moreover, inflammatory stimuli such as TNF-α and LPS suppressed PPARα activity, which could be restored by potent PPARα agonists.

**Conclusion:**

These findings suggest that PPARα activation modulates macrophage polarization and suppresses inflammatory signaling in response to periodontal bacterial antigens.

## Introduction

Periodontitis (PD) is a chronic inflammatory disease characterized by progressive destruction of tooth-supporting structures, including gingival tissue, periodontal ligament, and alveolar bone. It is primarily driven by dysbiotic microbial communities, with *Porphyromonas gingivalis* (Pg) recognized as a key pathogenic contributor. In addition to local tissue degradation and tooth loss, periodontitis has been increasingly linked to systemic inflammatory conditions, such as cardiovascular diseases and diabetes.^1,2^

Macrophages are pivotal in immune responses in the periodontium. Depending on the type of stimulation, they can polarize toward either a classically activated (M1) phenotype, which drives inflammation and tissue destruction by the secretion of pro-inflammatory cytokines such as tumor necrosis factor-alpha (TNF-α), or an alternatively activated (M2) phenotype, which supports inflammation resolution and tissue repair via mediators such as interleukin-10 (IL-10).^3,4^ *Porphyromonas gingivalis* lipopolysaccharide (Pg-LPS), a major virulence factor of *Pg*, activates macrophages predominantly via the NF-κB signaling cascade—a central inflammation regulator—leading to increased TNF-α production and subsequent periodontal tissue damage.^5^

CD36, a class B scavenger receptor, plays multifaceted roles in innate immunity and inflammation. In pathological contexts such as foam cell formation and atherosclerotic plaque development, CD36 contributes to pro-inflammation by facilitating oxidized lipid uptake, amplifying Toll-like receptor 2 (TLR2) signaling, and promoting cytokine production.^6-8^ Conversely, in tissue-reparative contexts, CD36 exerts anti-inflammatory effects by mediating efferocytosis, enhancing IL-10 secretion, and supporting transforming growth factor-beta 1 (TGF-β1) signaling.^9-13^ These contrasting functions suggest that CD36 may be a dynamic regulator of macrophage polarization. However, its specific role in Pg-LPS-induced macrophage responses remains poorly understood.

Fenofibrate, a clinically approved peroxisome proliferator-activated receptor-alpha (PPARα) agonist used in the treatment of dyslipidemia,^14^ has demonstrated anti-inflammatory properties across multiple disease models.^15,16^ Previous work from our group showed that fenofibrate attenuates periodontal inflammation and bone loss in Pg-induced ligature models,^17,18^ yet the molecular mechanisms responsible for this protective effect remain unclear. Notably, CD36 is a well-established transcriptional target of PPARα,^19^ with its expression regulated via PPAR response elements in the CD36 promoter. This regulatory axis connects lipid metabolism to immune modulation and macrophage function.

Given the central role of macrophages in periodontal pathogenesis and the emerging importance of the PPARα–CD36 axis in regulating inflammation, we hypothesized that PPARα activation by fenofibrate modulates macrophage polarization and suppresses TLR2/4–NF-κB-mediated inflammatory signaling. In this study, we investigate the immunomodulatory effects of fenofibrate on Pg-LPS-stimulated THP-1-derived macrophages, focusing on key markers of inflammation (TNF-α), resolution (IL-10), and polarization (CD36). Our goal is to elucidate the molecular pathways through which PPARα activation reshapes macrophage function in periodontitis.

## Methodology

### Cell culture and differentiation

Human monocytic THP-1 cells (ATCC) were cultured in RPMI 1640 medium (Invitrogen), supplemented with 10% heat-inactivated fetal bovine serum (FBS, Invitrogen), 10 mM HEPES (Gibco, #15630-056), 1 mM sodium pyruvate (Gibco, #11360-039), 2.5 g/L D-glucose (Merck), and 50 pM β-mercaptoethanol (Gibco, #31350-010). To induce macrophage differentiation, THP-1 cells were treated with 150 nM phorbol 12-myristate 13-acetate (PMA; Sigma, P8139) for 24 hours, followed by a 24-hour recovery period in PMA-free RPMI medium.

For experimental treatments, 1×10⁶ THP-1 monocytes were seeded in 6-well plates, differentiated as described above, and pretreated with fenofibrate (50 μM) or WY14643 (100 μM) for 24 hours. Cells were then stimulated with Pg-LPS (1 μg/mL) for an additional 24 hours, with or without the PPARα antagonist GW6471 (10 μM) concurrently with LPS.

### NF-κB reporter assay

THP-1 cells were transfected with a secreted embryonic alkaline phosphatase (SEAP) reporter plasmid driven by the ELAM-1 promoter, containing tandem MD-2/NF-κB and AP-1 binding sites (InvivoGen, San Diego, CA). Forty-eight hours post-transfection, cells were stimulated with Pg-LPS (1 μg/mL) for 24 hours, with or without fenofibrate (50 μM). SEAP levels, reflecting NF-κB/AP-1 activation, were quantified using Quanti-Blue reagent (InvivoGen), following the manufacturer’s instructions. Briefly, 20 µl cell culture supernatant was collected and combined with 180 µl of Quanti-Blue reagent in a 96-well plate, followed by incubation one hour at 37 °C. Absorbance at 492 nm was measured using a microplate reader (Vmax Kinetic Microplate, Molecular Devices).

C3H/TLR4^mut^ reporter cells, derived from TLR4-deficient murine embryonic fibroblasts and stably expressing an NF-κB-SEAP reporter (InvivoGen), were used as a TLR4-deficient negative control for normal reporter cells, which express TLR4 and respond to LPS. Cells were treated with LPS (1 μg/mL) or the TLR2 ligand Pam3CSK4 (100 μg/mL), with or without fenofibrate (50 μM), and SEAP activity was measured as previously described.

### Western blot analysis

Total cell lysates were prepared and subjected to SDS-PAGE, followed by Western blotting.^20^ Proteins were probed using the following primary antibodies: rabbit anti-TNF-α (Abcam, ab6671; 1:1000), rabbit anti-phospho-NF-κB (Cell Signaling, #3033; 1:500), rabbit anti-IL-10 (Abcam, ab34843; 1:1000), and rabbit anti-β-actin (Abcam, ab8227; 1:2000). Signal intensities were normalized to β-actin and quantified using ImageJ software (NIH, Bethesda, MD).

### Immunofluorescence microscopy

THP-1 monocytes were seeded at 1×10⁵ cells/well in 24-well plates with glass coverslips and differentiated into macrophages. Cells were fixed with 4% paraformaldehyde in PBS for 10 minutes, then blocked with 2% BSA in PBS. Primary antibodies (1:100 in 2% BSA-PBS) were applied overnight at 4 °C: anti-IL-10 (Abcam, ab955), anti-CD36 (Santa Cruz, sc-9099), and anti-NF-κB (Santa Cruz, sc-9154). After washing, Alexa Fluor 488-conjugated anti-rabbit IgG (Molecular Probes, A11034; 1:1000) was added for one hour at room temperature. Coverslips were mounted in Mowiol (Sigma) and imaged using a Zeiss LSM 780 confocal microscope.

### Flow cytometry

THP-1 cells (2.5×10⁶) were seeded in T25 flasks and differentiated using PMA as described. Following LPS stimulation (1 μg/mL) with or without fenofibrate (50 μM), cells were washed with PBS and detached using 5 mM EDTA. After blocking with cold PBS containing 5% heat-inactivated human serum and 0.1% sodium azide, cells were stained with fluorophore-conjugated monoclonal antibodies (BioLegend): FITC-anti-CD14, APC-anti-CD86, PE-anti-CD68, APC-anti-CD206, and APC-anti-CD163. A minimum of 50,000 events per sample were collected using a BD FACSAria cytometer, and data were analyzed with FlowJo (TreeStar Inc., San Carlos, CA).

### PPARα reporter assay

PPARα reporter cells (INDIGO Biosciences), stably expressing human PPARα (NR1C1) and a luciferase reporter, were seeded at 5×10^3^ cells/well in 96-well plates. Cells were treated with increasing concentrations of GW590735 (0–100 nM) or WY14643 (0–100 μM) to determine EC₅₀ values. Reporter cells were then exposed to TNF-α (0.5–5 ng/mL), LPS (0.25–1 μg/mL), or GW6471 (25-200 μM)+LPS (1 μg/mL) at the EC₅₀ concentration. Luminescence reflecting PPARα activity was measured using a luminometer according to the manufacturer’s instructions.

### Statistical analysis

Data are expressed as mean ± standard deviation (SD). Comparisons between two groups were analyzed using an unpaired Student’s *t*-test. One-way ANOVA followed by Tukey’s post hoc test was used for multiple group comparisons. A *p*-value < 0.05 was considered statistically significant. All statistical analyses were performed using GraphPad Prism (GraphPad Software, San Diego, CA). All sample numbers (n) represent biological replicates.

## Results

### PPARα activation decreases TNF-α expression in macrophages

TNF-α is a key pro-inflammatory cytokine predominantly produced by M1 macrophages and is central in mediating inflammatory tissue damage. In periodontitis, elevated TNF-α levels are strongly associated with inflammatory cell recruitment, extracellular matrix degradation, and alveolar bone resorption. TNF-α expression thus serves as a hallmark of M1 macrophage polarization and a key driver of periodontal tissue destruction.

Pg-LPS is a major virulence factor that potently stimulates pro-inflammatory responses in macrophages by activating the NF-κB signaling cascade.^5^ This activation leads to increased transcription of TNF-α and other inflammatory mediators, thereby exacerbating periodontal disease progression.

**Figure 1:**
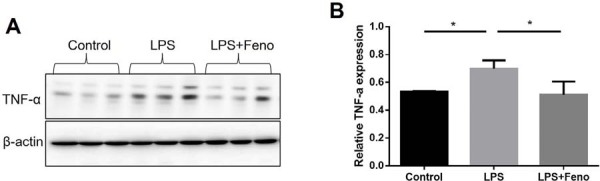
Fenofibrate suppresses TNF-α expression in Pg-LPS-stimulated THP-1 macrophages.

To investigate the anti-inflammatory potential of PPARα activation, THP-1-derived macrophages were stimulated with Pg-LPS in the presence or absence of fenofibrate, a selective PPARα agonist. As expected, Pg-LPS stimulation significantly increased TNF-α expression. However, co-treatment with fenofibrate markedly suppressed Pg-LPS-induced TNF-α levels, suggesting that PPARα activation attenuates inflammatory responses in macrophages (Figure 1A, 1B).

After reaching approximately 70% confluence, THP-1-derived macrophages were pretreated with fenofibrate (50 μM) or vehicle for 24 hours, followed by stimulation with Pg-LPS (1 μg/mL) for an additional 24 hours. TNF-α protein levels were assessed by Western blot (A) and quantified by densitometry (B). Each lane represents an individual biological replicate (mean±SD, n=4, *p<0.05).

### PPARα activation attenuates NF-κB signaling in macrophages

**Figure 2:**
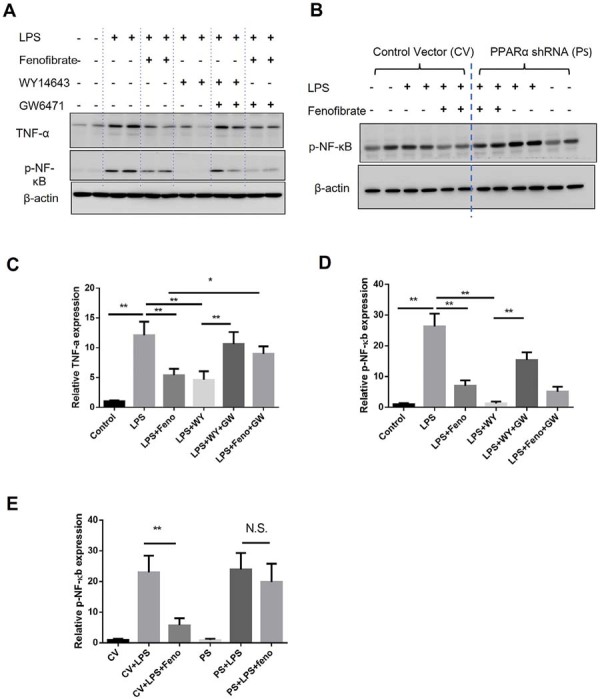
PPARα activation inhibits LPS-induced TNF-α expression and NF-κB signaling in THP-1 macrophages. (A) Representative Western blot analysis of TNF-α expression and NF-κB activation, indicated by phosphorylated NF-κB (p-NF-κB), in THP-1-derived macrophages. Cells were pretreated with PPARα agonists fenofibrate (50 μM) or WY14643 (100 μM) for 24 hours, followed by stimulation with Pg-LPS (1 μg/mL) for an additional 24 hours, in the presence or absence of the PPARα antagonist GW6471 (10 μM). (B) Representative Western blot of NF-κB activation in THP-1 macrophages transfected with PPARα-targeting shRNA and stimulated with Pg-LPS (1 μg/mL), with or without fenofibrate (50 μM). (C) Quantification of TNF-α expression from panel A. (D) Quantification of p-NF-κB expression from panel A. (E) Quantification of p-NF-κB levels from panel B. (mean±SD, n=4, *p<0.05, **p<0.01. N.S., not significant).

As TNF-α expression is regulated by TLR-mediated activation of the NF-κB signaling pathway, we next examined whether fenofibrate suppresses NF-κB activation in macrophages via PPARα activity. Phosphorylated NF-κB (p-NF-κB) is a key indicator of pathway activation. In THP-1-derived macrophages, Pg-LPS stimulation increased p-NF-κB levels (Figure 2A, 2D), consistent with enhanced TNF-α expression (Figure 2A, 2C). Fenofibrate treatment significantly reduced p-NF-κB levels, indicating inhibition of NF-κB activation (Figure 2A, 2D).

To further confirm the involvement of PPARα, we used WY14643, a selective PPARα agonist, which also reduced Pg-LPS-induced NF-κB phosphorylation and TNF-α expression. This effect was abolished by co-treatment with GW6471, a PPARα antagonist, confirming the specificity of PPARα-mediated signaling (Figure 2A, 2C, 2D). Additionally, a shRNA-mediated knockdown of PPARα reversed the inhibitory effects of fenofibrate on both NF-κB activation and TNF-α expression, further supporting the role of PPARα in mediating these anti-inflammatory responses (Figure 2B, 2E).

### Fenofibrate inhibits NF-κB activation via TLR2/4-dependent pathways

**Figure 3:**
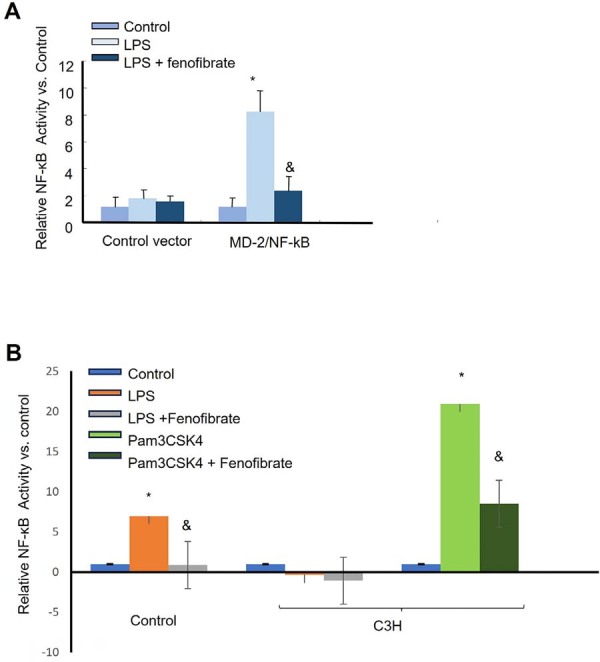
Fenofibrate suppresses NF-κB transcriptional activity via TLR4- and TLR2-mediated signaling pathways. (A) THP-1 cells were transfected with 1 μg of plasmid DNA encoding a SEAP reporter gene under the control of MD-2/NF-κB/AP-1 regulatory elements to generate stable reporter cells. Once quiescent, reporter cells were stimulated with Pg-LPS (1 μg/mL) in the presence or absence of fenofibrate (50 μM) for 24 hours. NF-κB transcriptional activity was quantified by measuring SEAP activity. (B) WT and C3H/TLR4 mutant reporter cells, stably expressing an NF-κB reporter construct, were stimulated with either LPS (1 μg/mL) with or without fenofibrate (50 μM) for 24 hours. C3H/TLR4 mutant reporter cells were also stimulated with the TLR2 ligand Pam3CSK4 (100 μg/mL) with or without fenofibrate (50 μM) for 24 hours. SEAP activity was measured to assess NF-κB activation (mean±SD, n=5. *p<0.05 vs. control group; & p<0.01 vs. LPS group).

Given that Pg-LPS activates NF-κB signaling primarily via TLR2/4 in coordination with Myeloid differentiation factor-2 (MD-2), we examined NF-κB activation using a luciferase reporter assay to dissect upstream signaling mechanisms. THP-1 reporter cells were transfected with NF-κB luciferase reporters along with MD-2 constructs. As shown in Figure 3A, Pg-LPS stimulation induced an 8.5-fold increase in NF-κB activity in cells expressing MD-2, which was reduced to 3.6-fold by fenofibrate treatment.

To further validate this hypothesis, we employed a C3H/HeJ murine macrophage reporter system carrying a mutant TLR4 gene, which renders cells unresponsive to LPS. In this system, Pg-LPS failed to activate NF-κB signaling, confirming the TLR4 dependency of LPS responsiveness in mice. However, stimulation with Pam3CSK4, a TLR2-specific ligand, robustly activated NF-κB, and this activation was significantly attenuated by fenofibrate (Figure 3B). These findings indicate that fenofibrate exerts anti-inflammatory effects by inhibiting both TLR2 and TLR4 pathways.

### Fenofibrate modulates macrophage polarization toward an anti-inflammatory phenotype

**Figure 4:**
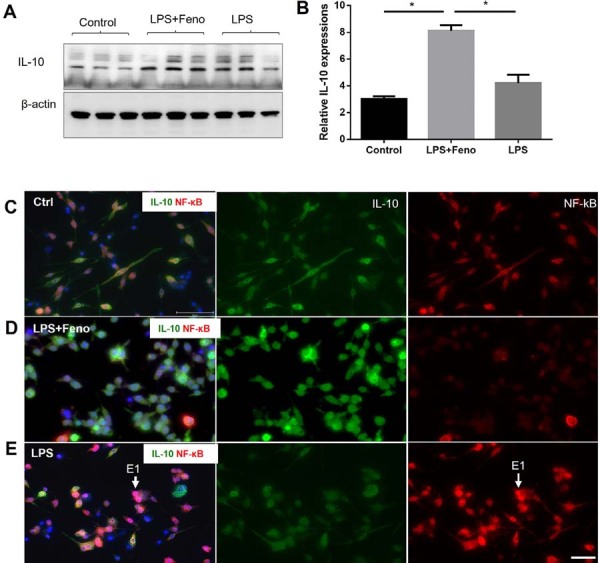
Fenofibrate enhances IL-10 expression and inhibits NF-κB nuclear translocation in Pg-LPS-stimulated THP-1 macrophages.

IL-10 is a potent anti-inflammatory cytokine secreted primarily by M2 macrophages. To determine whether fenofibrate also enhances anti-inflammatory responses, we measured IL-10 levels in THP-1 cells treated with fenofibrate. Fenofibrate treatment significantly increased IL-10 expression (1.0-fold increase compared with the LPS-only group) (Figure 4A, 4B, 4E), indicating promotion of anti-inflammatory macrophage activity.

(A) THP-1-derived macrophages were treated with fenofibrate (50 μM) for 48 hours, followed by stimulation with Pg-LPS (1 μg/mL) for an additional 24 hours. IL-10 protein levels were evaluated by Western blot. (B) Densitometric quantification of IL-10 expression based on western blot results. Each lane represents an individual biological replicate. (mean ± SD, n=4, *p<0.05). (C–E) Representative immunofluorescence analysis of IL-10 and NF-κB in THP-1 macrophages stimulated with Pg-LPS (1 μg/mL) for 24 hours, followed by treatment with or without fenofibrate (50 μM) for an additional 24 hours. In control cells, NF-κB remained cytoplasmic (C). Fenofibrate treatment increased IL-10 expression and inhibited NF-κB nuclear localization (D). Pg-LPS induced NF-κB nuclear translocation (E, arrows, E1). Red: NF-κB; Green: IL-10. Scale bar: 20 μm.

**Figure 5:**
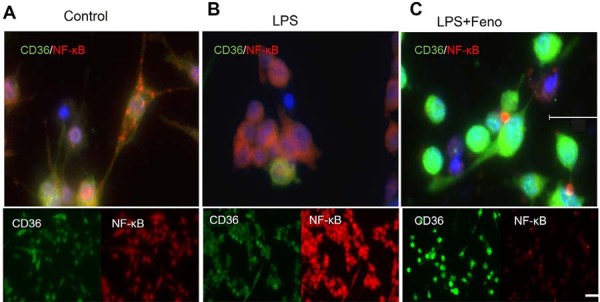
Fenofibrate enhances CD36 expression in THP-1 macrophages.

To further assess macrophage polarization, we analyzed CD36 expression, a surface marker associated with anti-inflammatory M2 macrophages and known to promote IL-10 secretion.^21^ Immunofluorescence staining revealed that fenofibrate treatment markedly increased CD36 expression in THP-1-derived macrophages (Figure 5A, 5B, 5C). In parallel, fenofibrate inhibited Pg-LPS-induced nuclear translocation of NF-κB (Figure 4C, 4D, 4E), suggesting that PPARα activation promotes an anti-inflammatory macrophage phenotype while suppressing pro-inflammatory signaling pathways.

Representative immunofluorescence staining was performed to assess NF-κB activation and CD36 expression in THP-1-derived macrophages. Cells were stimulated with Pg-LPS (1 μg/mL) for 24 hours in the presence or absence of fenofibrate (50 μM) for an additional 24 hours. In control cells, CD36 expression is normal (A). Pg-LPS stimulation decreased CD 36 expression (B), whereas fenofibrate significantly upregulated CD36 expression (C), indicating a shift toward an anti-inflammatory phenotype. Red: NF-κB; Green: CD36. Scale bar: 50 μm (upper panel), 20 μm (lower panel).

**Figure 6:**
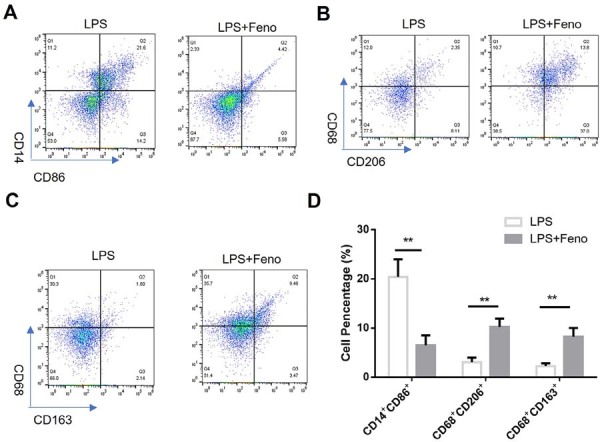
Fenofibrate modulates macrophage surface marker expression in Pg-LPS-stimulated THP-1 derived macrophages.

To assess the effects of fenofibrate on macrophage polarization, we analyzed canonical surface markers of M1 and M2 macrophages by flow cytometry. THP-1-derived macrophages stimulated with Pg-LPS exhibited significant increase in the CD14⁺CD86⁺ population, consistent with pro-inflammatory M1 activation. Fenofibrate treatment significantly reduced this M1 population (Figure 6A), indicating suppression of Pg-LPS-induced inflammatory polarization.

(A-C) Representative flow cytometry plots demonstrate the effects of fenofibrate on macrophage surface marker expression. THP-1-derived macrophages were stimulated with Pg-LPS (1 μg/mL) for 24 hours in the presence or absence of fenofibrate (50 μM), followed by staining and flow cytometric analysis. (A) Cells were stained with antibodies against CD14 and CD86, markers of pro-inflammatory (M1) macrophages. (B–C) Co-staining with M2-associated markers showed that fenofibrate increased the percentage of CD206^+^CD68^+^ (B) and CD68^+^CD163^+^ (C) macrophages, indicating a phenotypic shift toward an anti-inflammatory profile. (D) Quantification of surface marker expression for M1 and M2 populations. (mean±SD, n=4, **p<0.01).

Conversely, co-staining for anti-inflammatory M2 markers revealed that fenofibrate treatment increased percentages of CD68⁺ CD206⁺ (Figure 6B) and CD68⁺CD163⁺ (Figure 6C) macrophages, indicating a phenotypic shift toward an anti-inflammatory profile. Quantitative analysis confirmed that fenofibrate promoted coordinated upregulation of M2-associated markers while suppressing M1 surface expression (Figure 6D).

These results suggest that PPARα activation by fenofibrate reprograms macrophages toward an M2-like phenotype and attenuates Pg-LPS-induced M1 activation, contributing to the anti-inflammatory and immunomodulatory effects observed in this model.

### TNF-α and LPS suppress PPARα activity, reversed by PPARα agonists

**Figure 7:**
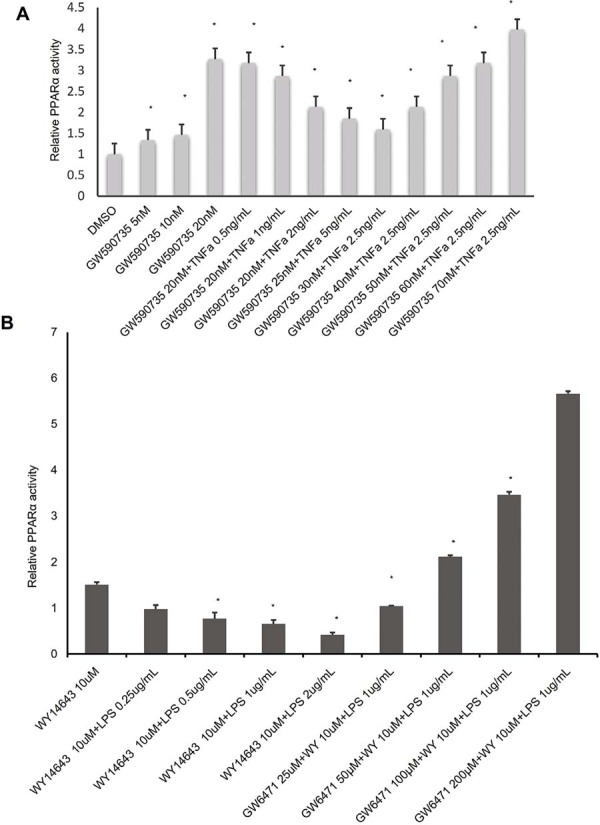
Pro-inflammatory stimuli suppress PPARα activity, which is restored by pharmacological agonists.

Both TNF-α and LPS are well-established stimulators of M1 macrophage polarization. To investigate whether they directly affect PPARα activity, we employed a PPARα reporter assay system. As shown in Figure 7A, PPARα activity was dose-dependently increased by the selective agonist GW590735. However, co-treatment with TNF-α caused a dose-dependent inhibition of PPARα activity, which was reversed by further dose escalation of GW590735.

Similarly, Figure 7B demonstrates that PPARα activity, initially stimulated by WY14643 at 10 μM, was dose-dependently suppressed by LPS. This suppression was reversed in a dose-dependent manner by increasing GW6471 concentrations. These findings suggest that pro-inflammatory stimuli such as TNF-α and LPS directly inhibit PPARα activity, and this inhibition can be overcome by sufficient activation via PPARα agonists.

Together, these data highlight PPARα as a critical modulator of macrophage polarization, integrating inflammatory signaling and metabolic regulation to balance pro- and anti-inflammatory phenotypes.

## Discussion

Our findings demonstrate that PPARα activation by fenofibrate is central in modulating macrophage polarization during Pg-LPS-induced inflammatory responses. Specifically, PPARα activation suppresses key pro-inflammatory features, including TNF-α expression and NF-κB signaling, while enhancing anti-inflammatory characteristics such as IL-10 production and upregulation of M2 surface markers. These results support a model in which PPARα functions as a transcriptional regulator, integrating inflammatory signaling and metabolic programming to shift macrophages from a classically activated M1 profile to an alternatively activated M2 state.

Macrophage polarization is an important determinant of immune responses in periodontitis. Persistent microbial stimulation drives macrophages toward a pro-inflammatory M1 phenotype, perpetuating tissue destruction. Our data show that fenofibrate significantly attenuates this process by reducing Pg-LPS-induced TNF-α expression and inhibiting NF-κB activation, both critical drivers of periodontal inflammation. Concurrently, fenofibrate enhanced IL-10 secretion and increased the prevalence of CD206⁺ and CD163⁺ macrophages—hallmarks of M2 polarization—supporting its role in promoting inflammation resolution and tissue repair.

While CD36 has been previously implicated in pro-inflammatory responses, particularly in foam cell formation and atherosclerosis via TLR2 signaling,^6,7^ our findings in Pg-LPS-stimulated macrophages reveal a contrasting role: CD36 expression was upregulated following fenofibrate treatment and was associated with enhanced IL-10 production, reduced NF-κB activation, and increased expression of M2 surface markers. These observations are consistent with recent studies demonstrating that CD36 supports anti-inflammatory functions in macrophages by facilitating efferocytosis, promoting fatty acid oxidation, and inducing mediators such as IL-10 and TGF-β1. Thus, although the role of CD36 may differ depending on cell type and microenvironment, our data support its function as an anti-inflammatory effector downstream of PPARα activation in the context of periodontal inflammation. Moreover, CD36 upregulated by fenofibrate treatment may also directly interact with TLR2 pathways, potentially reducing TLR2-mediated inflammation. This mechanism warrants further investigation in future studies.

We also observed that pro-inflammatory stimuli such as TNF-α and LPS suppress PPARα transcriptional activity in macrophages, consistent with the notion that inflammation reinforces M1 polarization by silencing nuclear receptor-driven resolution pathways. This suppression was reversed by higher concentrations of PPARα agonists, further underscoring the therapeutic potential of pharmacologically activating PPARα to reprogram macrophage phenotypes in inflammatory diseases.

Therefore, our study identifies PPARα as a pivotal regulator of macrophage polarization in response to Pg-LPS, highlighting its role in suppressing pro-inflammatory signaling while promoting anti-inflammatory and tissue-restorative phenotypes. Although CD36 contributed to this phenotypic shift, its role appears to be context-dependent and likely secondary to the broader effects of PPARα activation. Future studies employing CD36 loss-of-function approaches are necessary to delineate its precise contribution to the anti-inflammatory actions of PPARα in macrophages.

## Conclusion

These findings have broad implications for chronic inflammatory diseases such as periodontitis, in which sustained M1 polarization contributes to progressive tissue destruction. Targeting PPARα offers a promising strategy to modulate macrophage function and restore immune balance in the inflamed periodontium.

Luciferase-based reporter assays were utilized to evaluate PPARα transcriptional activity under pro-inflammatory conditions. (A) PPARα reporter cells were treated with increasing concentrations of the selective PPARα agonist GW590735 (5–20 nM), in the presence or absence of recombinant TNF-α (0–2.5 ng/mL). TNF-α induced a dose-dependent suppression of GW590735-induced PPARα activity, which was progressively restored by escalating GW590735 concentrations (30–70 nM). (B) Confluent PPARα reporter cells were stimulated overnight with WY14643 (10 μM), followed increasing concentrations of Pg-LPS (0–2 μg/mL). At 1 μg/mL LPS, titration of GW6471 (25 μM–200 μM) reversed LPS-induced suppression of PPARα activity. Luciferase activity was quantified using a luminometer (mean±SD, n=4, *p<0.05).
